# Draft Genome Sequence of a Tetrabromobisphenol A–Degrading Strain, *Ochrobactrum* sp. T, Isolated from an Electronic Waste Recycling Site

**DOI:** 10.1128/genomeA.00680-16

**Published:** 2016-07-21

**Authors:** Zhishu Liang, Guiying Li, Taicheng An, Guoxia Zhang, Ranjit Das

**Affiliations:** a State Key Laboratory of Organic Geochemistry and Guangdong Key Laboratory of Environmental Protection and Resources Utilization, Guangzhou Institute of Geochemistry, Chinese Academy of Sciences, Guangzhou, China; b Institute of Environmental Health and Pollution Control, School of Environmental Science and Engineering, Guangdong University of Technology, Guangzhou, China; c University of Chinese Academy of Sciences, Beijing, China; d Southern Medical University, Guangzhou, China

## Abstract

*Ochrobactrum* sp. T was previously isolated from a sludge sample collected from an electronic waste recycling site and characterized as a unique tetrabromobisphenol A (TBBPA)–degrading bacterium. Here, the draft genome sequence (3.9 Mb) of *Ochrobactrum* sp. T is reported to provide insights into its diversity and its TBBPA biodegradation mechanism in polluted environments.

## GENOME ANNOUNCEMENT

Environmental contamination by tetrabromobisphenol A (TBBPA), the most widely used brominated flame retardant, is a matter of pollution concern worldwide (1). Thus, there is a heightened need to elucidate its environmental fate. As known, TBBPA-degrading bacteria play an important role in the remediation of TBBPA-polluted sites, which attract much attention from researchers (2). A strain capable of simultaneous debromination and aerobic mineralization of TBBPA was previously isolated from sludge collected from Guiyu Town, southern China, and characterized as *Ochrobactrum* sp. T (3). Its genome sequence was determined to gain insights into its TBBPA biodegradation mechanism and the genetic features of the species within the family of *Brucellaceae*.

The whole-genome sequence was performed using the Illumina MiSeq system at Shanghai Majorbio Bio-pharm Technology Co., Ltd. (Shanghai, China). A total of 3,348,572 paired-end reads were generated with an average insert size of 350 bp and 511.48-fold coverage. The sequences obtained were trimmed by setting the quality score limit at 20 and discarding reads <25 bp, and assembled with SOAPdenovo version 2.04 (4) (at a *k*-mer length of 31). The final draft genome of strain T yielded 48 contigs with an *N*_50_ of 419,558 bp and a total assembled length of 3,938,898 bp. The NCBI Prokaryotic Genome Annotation Pipeline (PGAP) and RAST server (5) were used to annotate the genome sequence.

The draft genome sequence length was 982,890 bp with a G+C content of 57.8 %. The genome contains 3,877 putative open reading frames (with an average size of 872 bp) predicted by Glimmer version 3.02 (6), giving a coding intensity of 85.8%. Together, the genome contains 3,629 protein coding sequences (CDSs), 57 pseudogenes, 35 RNA-encoding genes, and four noncoding RNAs (ncRNAs), which were identified with the help of Barrnap and tRNAscan-SE (7). Of the CDSs, 80.7% can be assigned to clusters of orthologous groups, with amino acid transport and metabolism as the most abundant class. Moreover, 54.4% can be annotated into 2,005 KEGG orthologous groups using KAAS (8), involving 176 metabolic pathways.

RAST annotation showed that *Brucella suis* S2 (score 507), *Brucella melitensis* M111 (score 451), and *Ochrobactrum intermedium* LMG 3301 (score 450) were the closest neighbors of strain T. RAST also showed that many genes possibly involved in the metabolism of aromatic compounds were in the strain T genome. For example, genes coding 3-oxoadipate CoA-transferase subunit B (EC 2.8.3.6), beta-ketoadipyl CoA thiolase (EC 2.3.1), and beta-ketoadipate enol-lactone hydrolase (EC 3.1.1.24), which are involved in the degradation pathway of chloroaromatic compounds, were present in the genome. Genes possibly responsible for TBBPA degradation were analyzed. The results revealed that genes encoding halogenated organic compound degradation included one haloalkanoic acid dehalogenase and four haloacid dehalogenases. Moreover, three HAD family hydrolases and seven dioxygenases were also present in the genome. Thus, the strain T genome sequence and its curated annotation are important assets for better understanding the physiology of the strain and the microbial mechanisms of TBBPA biodegradation.

### Nucleotide sequence accession numbers.

This whole-genome shotgun project has been deposited at DDBJ/ENA/GenBank under the accession number LXEK00000000. The version described in this paper is the first version, LXEK01000000.
